# The Role of lncRNA Crosstalk in Leading Cancer Metastasis of Head and Neck Squamous Cell Carcinoma

**DOI:** 10.3389/fonc.2020.561833

**Published:** 2020-10-02

**Authors:** Yu Wang, Sinan Wang, Yu Ren, Xuan Zhou

**Affiliations:** ^1^Department of Maxillofacial and Otorhinolaryngological Oncology, Tianjin Medical University Cancer Institute and Hospital, Tianjin, China; ^2^Key Laboratory of Cancer Prevention and Therapy, Tianjin Cancer Institute, Tianjin, China; ^3^National Clinical Research Center of Cancer, Tianjin, China; ^4^Department of Gastroenterology and Hepatology, Tianjin Medical University General Hospital, Tianjin, China; ^5^Tianjin Gastroenterology and Hepatology Institute, Tianjin Medical University, Tianjin, China; ^6^Tianjin Research Center of Basic Medical Science, Tianjin Medical University, Tianjin, China

**Keywords:** head and neck squamous cell carcinoma, long non-coding RNA, metastasis, EMT, invasion

## Abstract

Head and neck squamous cell carcinoma (HNSCC) is the sixth most common type of human malignancy. For decades, research into HNSCC invasion and metastasis has been dedicated to the study of protein-coding genes. Along with whole-genome and transcriptome sequencing development, long non-coding RNA (lncRNA) has attracted greater attention. Compelling evidence has proven the critical role of lncRNAs in the occurrence and development of HNSCC by means of epigenetic modifications, regulation of gene transcription, and post-transcription level. More importantly, crosstalk between lncRNAs and microRNAs was recently proven to regulate HNSCC metastasis through EMT modification. Based on these, this review summarizes the critical roles of lncRNAs in HNSCC metastasis and the crosstalk between lncRNAs and microRNAs as well as the detailed regulatory mechanism of the interaction. Thus, a deeper understanding of the lncRNA network in cancer metastasis is finally uncovered in order to provide a rationale and innovative concepts toward new therapeutic strategies for the highly metastatic HNSCC.

## Introduction

Head and neck squamous cell carcinoma (HNSCC) is the sixth most common type of human malignancy and involves carcinoma of several anatomic sites, such as lip, oral cavity, pharynx (nasopharynx, oropharynx, hypopharynx), and larynx, with an annual incidence of ~500,000 (1). Even through systemic therapeutic strategies have developed, the 5-years overall survival (OS) of HNSCC patients is hardly satisfying (2). Evidence has shown that the relatively poor prognosis and high recurrence of HNSCC are mainly due to the high rate of local invasion and distant metastasis (3). Consequently, it is essential to explore the detailed molecular mechanisms involved in cancer metastatic cascade so as to promote the development of target therapy and improve the overall survival of HNSCC.

Experimental and clinical studies have attempted to establish the biological basis of this metastasis cascade. Mountains of evidence highlight the irreplaceable role of long non-coding RNA (lncRNA) in cancer metastasis, including HNSCC (4, 5). Such transcripts are widely validated not to produce functional proteins, but regulate gene expression at multiple levels and participate in cancer evolution and development (6). More importantly, unique cross-regulation between lncRNA and miRNA was recently mentioned, and emerging evidence shows that such crosstalk has a great effect on human cancer metastasis, partially through EMT regulation (7). In this review, we summarize the correlation between lncRNA and EMT mediation and highlight the leading role of lncRNA/miRNA crosstalk in the metastasis of HNSCC.

## LncRNAs Involved in HNSCC Invasion and Metastasis

LncRNAs are a heterogenous group of RNAs containing more than 200 nucleotides and recently involved in many biological processes (8). Their number is significantly larger than that of protein-coding genes and can act in a cis and/or in trans manner during the development of human cancers (9). They have well-defined subcellular sites, mainly concentrated in the nucleus and involved in the regulation of chromatin and chromosomal conformation (10).

In the past few years, there has been a paradigm shift in the understanding of non-coding RNAs (ncRNAs) and their role in cancer biology (11, 12). Due to the discovery of alternative splicing in 1970s, a major focus in various pathological and physiological processes shifted to the role of proteins and protein-coding RNAs and mutations as prominent mechanisms in disease etiology and pathophysiology. However, in 1977, the discovery of introns and ribozymes suggested the role of ncRNA as a regulatory molecule (13). Since then, more and more research focuses on lncRNAs, which are identified as playing critical roles in cancer metastasis regulation. LncRNAs are not only responsible for tumor proliferation, cell death regulation, and angiogenesis (14–16), but also for the invasion and metastasis of HNSCC (17–19). These lncRNAs described as potential transfer regulators are shown in Table 1.

**Table 1:** lncRNAs in HNSCC metastasis.

**LncRNA**	**Genome**	**Expression**	**Biological function and mechanism**	**References**
*NEAT1*	11q13.1	- Upregulated in OSCC; -Overexpressed in LSCC	- Promoted cell motility of OSCC by sponging miR-365; - Regulated CDK6 in LSCC, mediated by miR-107	(20–22)
*HOTAIR*	12q13.13	- Overexpressed in OSCC and LSCC and correlated tumor metastasis; - Increased level of HOTAIR and miR-21 in the blood of patients with LSCC were associated with T classification and lymph node metastasis	- Promoted tumor metastasis through EZH2 recruitment and E-cadherin silencing in OSCC; - Triggered metastasis in OSCC through modulation of EMT; - Promoted OSCC metastasis through miR-326-MTA2 axis	(17, 19, 23–25)
*HOTTIP*	7p15.2	- Overexpressed in TSCC samples and associated with clinical stage, distant metastasis, and OS rate	- HOTTIP silencing repressed tumor cell growth and resulted in a great rise of miR-124-3p and E-cadherin expression and a distinct fall of HMGA2, β-catenin, and c-Myc protein levels	(26, 27)
*UCA1*	19p13.12	- Overexpressed in TSCC samples; - Potentially prognostic indicator of lymph node metastasis	- Promoted cell migration and invasion by targeting miR-143-3p in OSCC; - Modulated TGF-β induced EMT and OSCC invasion through JAG1/Notch; - Contributed to OSCC progression by regulating the WNT/β-catenin signaling pathway	(28–32)
*MALAT1*	11q13.1	- Overexpressed in LSCC and OSCC samples, especially in metastatic TSCC with cervical lymph node metastasis	- Potentiated metastasis of TSCC via miR-140-5p/PAK1 axis modulation; - Modulated TSCC metastasis partially through the regulation of SPRR; - Mediated the pro-metastatic role of STAT3 in HNSCC via interaction with miR-30a; - Promoted OSCC metastasis by inducing EMT	(20, 33–36)
*H19*	11p15.5	- Overexpressed in patients with LSCC and positively correlated with cervical lymph node metastasis	- Promoted TSCC metastasis through β-catenin/GSK3β/EMT axis; - Facilitated TSCC invasion via sponging miR-let-7	(37–39)
*NAG7*	3p25.3	- Downregulated in NPC samples and positively correlated with cervical lymph node metastasis	- Promoted NPC invasion and metastasis via regulation of ERalpha and JNK2/AP-1/MMP1 signaling pathways	(40, 41)
*NKILA*	20q13.31	- Downregulated in TSCC tissues	- Attenuated migration and invasion of TSCC via EMT suppression	(42)
*LINC00467*	1q32.3	- Overexpressed in HNSCC and positively correlated cell motility and EMT process	- Raised USP48 expression through miR-299-5p regulation	(43)
*KTN1-AS1*	14q22.3	- Upregulated in HNSCC tissues	- Promoted cell proliferation, migration, invasion through miR-153-3p sponge in HNSCC; - Aberrant expression of SNAI1 and ZEB2 mediated the role of KTN1-AS1 due to miR-153-3p exhibition	(44)
*RC3H2*	9q33.2	- Overexpressed in OSCC tissues and the FISH assay verified the cytoplasm location	- Promoted OSCC proliferation, invasion, metastasis and increased level of EZH2 and H3K27Me3 expression; - Served as ceRNA sponging miR-101-3p and targeted EZH2	(45)
*AC091729.7*	Chromosome 7	- Upregulated and closely connected with the OS of the sinonasal squamous cell carcinoma (SNSCC) patients	- Served as a novel biomarker and latent curative target in SNSCC, through SRSF2 combination	(46)
*ADAMTS9-AS2*	3p14.1	- Significantly upregulated in TSCC tissues from patients with lymph node metastasis and is closely associated with poor prognosis	- ADAMTS9-AS2 knockdown led to the inhibition of cell migration and invasion and reversed TGF-β1 induced EMT; - Shared the miRNA response elements (MREs) of miR-600 with EZH2	(47)

### LncRNA HOTAIR

LncRNA HOX transcript antisense RNA (HOTAIR) is one of the most well-studied oncogenic lncRNAs, originally characterized as a regulator of the HOX gene family, helping to control cellular identity (48). The 5′ terminal of HOTAIR can be combined with chromatin modified complex, and the 3′ terminal can bind to histone demethylase I complex. Therefore, HOTAIR regulates methylation or demethylation of H3K4me2 at the H3K27 site, which is involved in proliferation (49), apoptosis (50), and metastasis (51) of tumor cells. It was found that the expression of HOTAIR is increased in many subtypes of HNSCC. Compared with the normal oral epithelial cell lines, HOTAIR in OSCC Cal-27 and UM-1 cell lines increased significantly (52). In addition, *in vitro* experiments demonstrate that, compared with low invasiveness, HOTAIR in the invasive oral squamous cell carcinoma cell lines is upregulated. Moreover, knocking down HOTAIR expression levels globally inhibits cell proliferation, migration, and invasion (53). Furthermore, HOTAIR is confirmed to promote HNSCC invasion and metastasis (23) and triggers the EMT process through EZH2/H3K27me3 recruitment, which is proven to be negatively associated with clinical outcomes in HNSCC patients (54).

### LncRNA UCA1

In tongue squamous cell carcinoma (TSCC), the expression level of lncRNA urothelial carcinoma antigen 1 (UCA1) is significantly increased and correlated with lymph node metastasis. In addition, the expression of UCA1 in lymph node metastasis is higher than that in the primary tumor. In a cell culture of TSCC, the overexpression of UCA1 promotes cell migration but has little effect on cell proliferation (32). Furthermore, UCA1 is also revealed to attenuate cell growth and metastasis of OSCC cell lines *in vitro* and *in vivo*, through WNT/β-catenin activation (28). Consequently, it is suggested that UCA1 may promote the metastasis of cancer cells and may be a prognostic indicator of lymph node metastasis in HNSCC.

### LncRNA MALAT1

Abnormal expression of lncRNA metastasis–associated lung adenocarcinoma transcript 1 (MALAT1) is reported in multiple human cancers, including prostate cancer (55), colorectal cancer (56), hepatocellular carcinoma (57), and HNSCC (33, 34). Fang et al. reported globally increased MALAT1 expression levels in TSCC (32), especially in those with lymph node metastasis (35). DNA microarray analysis shows that MALAT1 significantly increases TSCC cell motility through regulating LAYN, CCT4, CTHRC1, and FHL1 expression levels, which are small proline-rich protein (SPRR) members (35). In parallel, Zhou and colleagues reveal that MALAT1 is significantly associated with poor prognosis in patients with OSCC and could promote invasion and metastasis of OSCC by means of EMT activation (34). In addition, by means of ChIP-PCR and RIP-PCR analysis, we also reveal that STAT3 may accelerate EMT progression and cancer metastasis through interaction with the MALAT1/miR-30a axis (33).

### LncRNA H19

LncRNA H19 is described as participating in the metastasis of various cancers. In TSCC, H19 is demonstrated to be upregulated in the tumor tissue compared with adjacent samples. Furthermore, the expression level of H19 in metastatic tumor is significantly higher than in non-metastatic tumor. Subsequently, H19 is demonstrated to function as ceRNA to sponge let-7a, resulting in HMGA2 enhancement, and finally, increasing the capacity of TSCC invasion and metastasis (37). H19 is also found to be overexpressed in nasopharyngeal carcinoma (NPC) and to promote NPC cell invasion capacity via E-cadherin silencing and miR-630/EZH2 regulation (58). Mechanistically, Wu T. and colleagues report that H19 is overexpressed in laryngeal squamous cell carcinoma (LSCC) and accelerates LSCC tumor progression through miR-148a-3p attenuation and DNMT1 enhancement (39).

## LncRNAs Regulate HNSCC Cell Motility via EMT Mediation

The main regulating factors of EMT include the EMT effect factor, EMT core regulating factor, and EMT induction factor (59). EMT effect factors are usually proteins that define epithelial or mesenchymal properties, such as E-cadherin, α- Catenin, γ-Catenin, Vim, and Fibronectin, which promote cell migration and invasion during EMT. Among them, E-cadherin is considered to be the leading force (60). The core regulatory factors of EMT are composed of transcription factors, including Snail-1, Snail-2, ZEB1, ZEB2, Twist-1, and Twist-2 as well as the newly discovered pair-related homeobox transcription factor 1 (Prrx1), which regulates the EMT process through E-cadherin mediation (60). Moreover, EMT inducers consist of several signaling pathways, including TGF-β/Smad, Wnt/β-catenin, Notch, and GF receptor signaling cascade. Most importantly, the TGF-β/Smad signaling pathway appears to be the major activator of EMT. In addition, tumor inflammation, and the hypoxia microenvironment also serve basic roles in EMT promotion.

**Figure 1:**
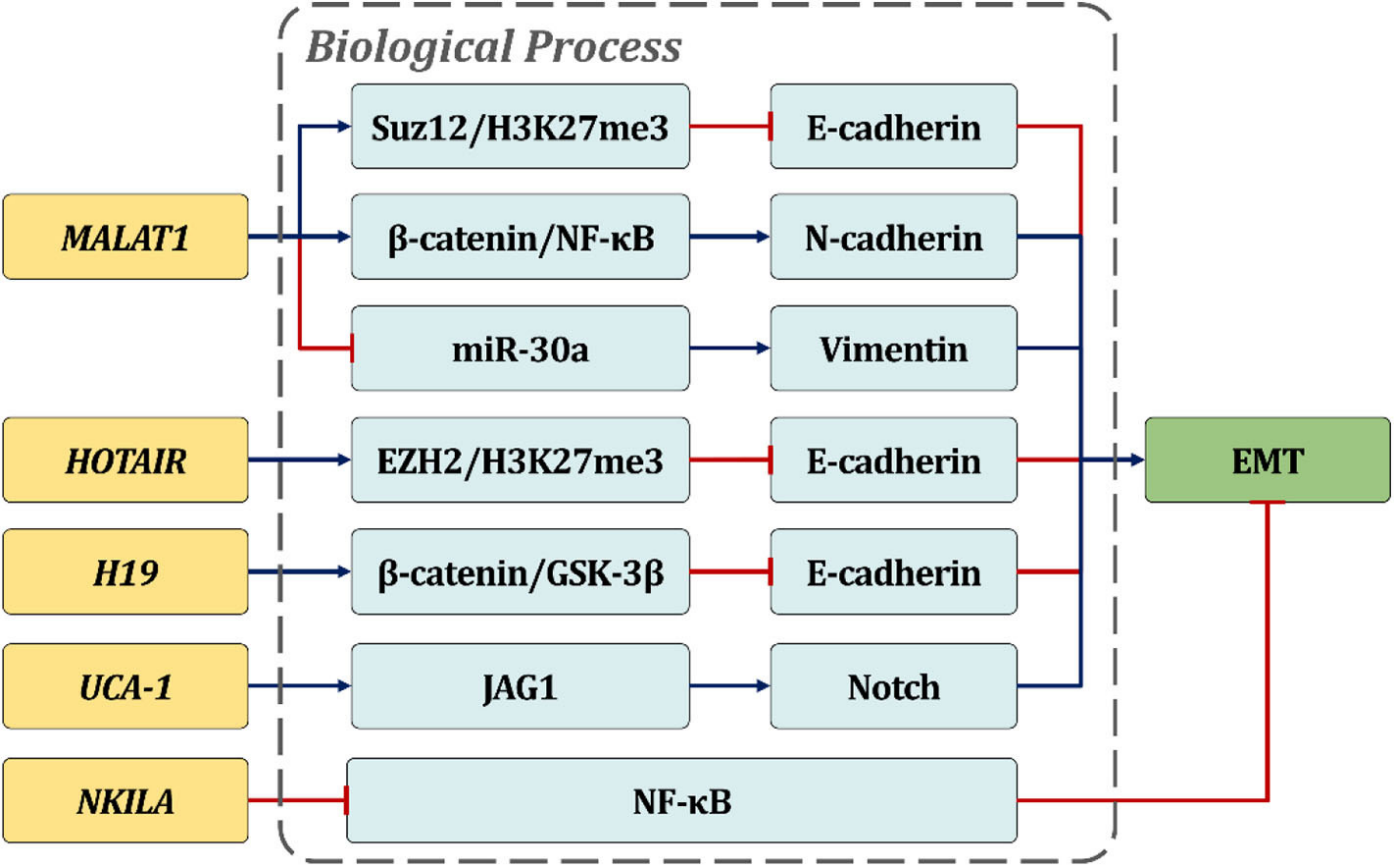
LncRNAs regulate HNSCC cell motility via EMT mediation. The text in the boxes depicts the potential pro/anti-EMT mechanisms of lncRNAs in HNSCC.

Recently, it is confirmed that EMT is also regulated by post transcription factor lncRNA, which plays its role through regulatory effectors, transcription factors, and signal transduction pathways (61). Unlike microRNAs, which repress target gene expression levels post-transcriptionally, functional lncRNAs may influence the EMT process during cancer metastasis by regulating gene expression at different levels, including chromatin modification, transcription, and post-transcriptional processing (Figure 1). MALAT1 is widely confirmed as one of the most well-studied oncogenic lncRNAs that is confirmed to be involved in the EMT process. Fan Y. and colleagues illustrate that TGF-β overexpression in the tumor microenvironment could induce cancer metastasis through EMT regulation and validate MALAT1 as an important mediator of TGF-β related EMT (62). Mechanistically, MALAT1 is then proven to promote EMT through Suz12 recruitment, which acts as an H3K27 methyltransferase binding E-cadherin promoter and inhibiting its expression in a PRC2-dependent manner. Moreover, subsequent ChIP-PCR and luciferase reporter assays show that STAT3 might bind to the MALAT1 promoter region and transcriptionally activate its expression in order to induce EMT and accelerate HNSCC metastasis (33). In OSCC, MALAT1 is also reported to play oncogenic roles in EMT-related cancer metastasis. By means of siRNA, MALAT1 is validated to be required for maintaining EMT-mediated cell migration and invasion. MALAT1 knockdown significantly suppressed the expression levels of N-cadherin and Vimentin, but raised E-cadherin *in vitro*. Meanwhile, both cytoplasm and the nucleus NF-κB/β-catenin axis is significantly triggered after MALAT1 elevation. It is noteworthy that targeting MALAT1 globally inhibits the proliferation capacity of TSCCA-induced xenograft tumor, suggesting MALAT1 as an important prognostic factor of OSCC and a satisfactory target with therapeutic potential. Furthermore, MALAT1 also acts as a transcriptional regulator within the regulation of activating the Wnt/β-catenin signaling pathway (63). In addition to MALAT1, there are other lncRNAs proven to participate in EMT regulation, such as HOTAIR and H19. In OSCC, a significant negative correlation between HOTAIR and E-cadherin expression levels is found in both tumor tissues and cell lines. Meanwhile, HOTAIR is validated to trigger E-cadherin silencing through the recruitment of EZH2 and H3K27me3 in the promoter region of E-cadherin (23), indicating that HOTAIR might regulate OSCC metastasis in an epigenetic manner. On the other hand, compared with matched normal tissues, the expression of H19 is upregulated in TSCC specimens and significantly correlated with lymph node metastasis. Subsequently, H19 attenuation significantly suppresses cell motility *in vitro* through activation of β-Catenin/GSK3β/E-cadherin signaling. In addition, animal models show that H19 inhibition significantly impairs tumor progression and lung metastasis (38). Apart from those prometastatic lncRNAs, lncRNA NKILA is validated to inhibit the migration and invasion of OSCC (42). Mechanistic study shows that NKILA inhibits the phosphorylation of IκBα and NF-κB activation as well as the induction of the EMT process. An *in vivo* experimental metastasis model also demonstrates that NKILA inhibits lung metastasis of NOD/SCID mice with TSCC tumors, suggesting NKILA as a potential predictor for OS and distant metastasis in patients with TSCC.

## LncRNA/Microrna Interaction in HNSCC Metastasis

**Figure 2:**
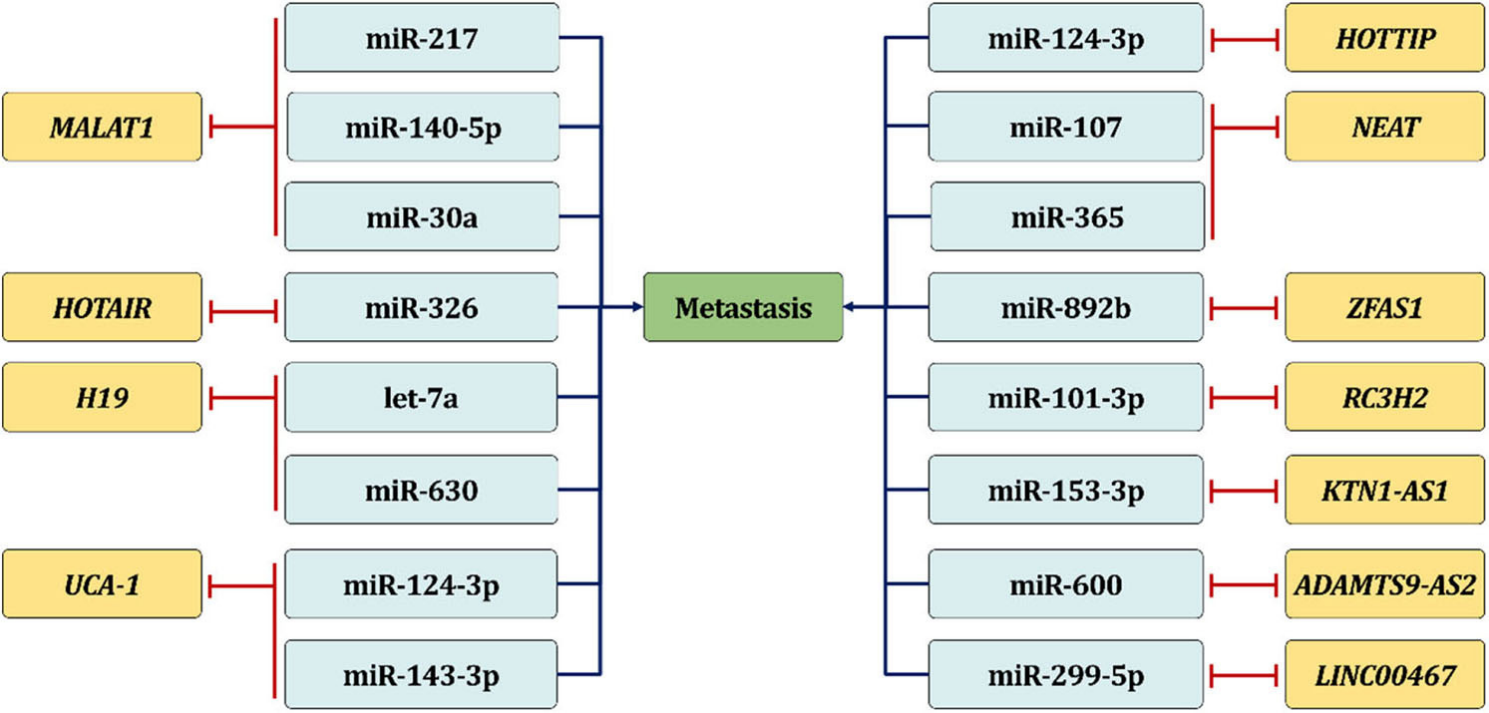
LncRNA/microRNA interaction in HNSCC metastasis. LncRNA can serve as ceRNA in order to bind certain miRNA to isolate the miRNA from its target mRNA, thereby promoting HNSCC metastasis.

During ncRNA crosstalk, on the one hand, the stability of lncRNA can be affected due to coaction with specific miRNA. On the other hand, lncRNA, also known as competitive endogenous RNA, could bind certain miRNAs to isolate the miRNA from its target mRNA, thereby antagonizing miRNA's function (Figure 2). The tumor suppressor miR-217 is reported to inhibit MALAT1 through the Ago2-mediated pathway in order to inhibit EMT-related metastasis through upregulating E-cadherin and N-cadherin suppression (64). Similarly, the recruitment of miR-30a also reduces the stability of MALAT1 in HNSCC in order to inhibit the invasion capacity of tumor cells (33). Additionally, MALAT1 knockdown is also seen to completely suppress tumor progression through miR-140-5p elevation and PAK1 inhibition, both *in vitro* and in TSCC-induced xenograft tumors (36). Another lncRNA H19 is also widely mentioned in lncRNA/miRNA interaction. Kou N. and colleagues illustrate that H19 can act as ce-RNA to sponge let-7a, leading to the accumulation of metastasis regulator HMGA2, which is enriched in TSCC tissues and cell lines. Intriguingly, let-7a suppression significantly rescues the weakened tumor cell motility induced by sh-H19. These findings demonstrate that the H19/let-7a crosstalk plays a critical role in TSCC migration and invasion (37). Meanwhile, H19 is validated to regulate EZH2 by miR-630 silencing, which is a repressor of EZH2 and interacts with H19 in a sequence-specific manner, to inhibit the expression level of E-cadherin and eventually accelerate the invasion and metastasis of NPC (58). Other examples involving lncRNA ZFAS1 (65) and lncRNA UCA1 (29, 30) can also function as ceRNA during EMT-related cancer metastasis.

## Perspectives

LncRNA is constitutively deregulated during the progression and development of human cancers and globally suggested as a critical regulator in tumor cell motility. At present, the understanding of lncRNA in HNSCC metastasis remains confused and ambiguous, and there is little information about the function of lncRNA in HNSCC, which needs further research. This review provides a comprehensive study of the expression profile of lncRNA in HNSCC and summarizes the control of ncRNA crosstalk on the EMT process, emphasizing the leading influence of lncRNA crosstalk in the metastasis of HNSCC. Even so, a great deal of work is still urgently required to characterize the complex ncRNA networks that contribute to HNSCC metastasis, and it is necessary to carry out further research to clarify the relationship between lncRNA and miRNA in order to seek better treatment strategies.

## Consent for Publication

All authors agreed to the publication of this review.

## Author Contributions

YW and SW contributed to conception, drafting, interpretation, and manuscript revision. XZ and YR contributed to interpretation and manuscript revision. All authors provided final approval and agreed to be accountable for all aspects of the study.

## Conflict of Interest

The authors declare that the research was conducted in the absence of any commercial or financial relationships that could be construed as a potential conflict of interest.
